# Opportunistic random blood glucose screening among professional drivers in northeastern Bangladesh: Assessing undiagnosed diabetes and health awareness

**DOI:** 10.1371/journal.pgph.0004828

**Published:** 2025-06-24

**Authors:** Md Sakil Arman, Md. Nayem Sarker, Jawad Ahmed, Aporajita Das Trisha, Sadia Rahman, Md. Monjurul Haq Shakib, Towhidul Alam, Zafrul Hasan

**Affiliations:** Department of Biochemistry and Molecular Biology, Shahjalal University of Science and Technology, Sylhet, Bangladesh; Nancy Angeline Gnanaselvam, St John’s Medical College, INDIA

## Abstract

Diabetes remains a silent epidemic in underrepresented high-risk groups like professional drivers, highlighting the urgent need for informed health policies and targeted interventions. This study aimed to assess the prevalence of undiagnosed diabetes and related health awareness among professional drivers in northeastern Bangladesh using opportunistic random blood glucose (RBG) testing to address knowledge gaps and inform health policy. A cross-sectional study was conducted on 1,454 participants enrolled between February 5, 2024 and July 27, 2024, using a consent-based questionnaire, anthropometric measurements, and RBG testing with a glucometer. Diabetes awareness was assessed using pre-tested questionnaires, while the prevalence of diabetes and associated factors were evaluated using Mann-Whitney U tests, Welch ANOVA, and Spearman correlation analysis. A total of 2.20% of the driver population were found to have undiagnosed diabetes. RBG levels differed significantly across regions. Middle age (7.63%) and overweight (3.77%) groups exhibited the highest prevalence of undiagnosed diabetes. Confounding variables such as BMI (r = 0.22, p < 0.0001), age (r = 0.19, p < 0.0001), and sleep duration (r = -0.05, p = 0.04) were significantly associated with glucose levels, indicating potential risk factors for diabetes. The obese group (AOR: 3.04, 95% CI: 0.81–11.46) and overweight group (AOR: 1.81, 95% CI: 0.83–3.99) were 3.04 and 1.81 times more likely, respectively, to develop diabetes compared to the healthy weight group. Participants with less than 7 hours of sleep (AOR: 1.13, 95% CI: 0.46–2.75) were also at greater risk. Co-morbidities and a family history of diabetes were also significantly associated with elevated RBG levels. Overall, this study highlights the regional and behavioral disparities influencing the development of diabetes risk among professional drivers, a population often neglected in health policy. It underscores the need for health education and large-scale RBG testing to improve awareness and alert policymakers in formulating effective health guidelines.

## Introduction

Diabetes is a significant public health concern in lower and middle-income countries (LMICs), affecting an estimated 80% of the adult population [1]. In South Asian countries, diabetes is considered the seventh leading attributable risk factor [2], contributing to severe health outcomes such as premature mortality [3], cardiovascular diseases [4,5], diabetic retinopathy [6], and increased susceptibility to tuberculosis, foot infections, and COVID-19 severity [7,8], thereby placing enormous strain on their already fragile health systems [9–11].

In Bangladesh, diabetes presents a similar public health burden. The number of adults living with diabetes was estimated at 13.14 million in 2021 and is projected to nearly double by 2045 if current trends continue [1,12]. A nationwide survey conducted in 2017 reported a 13% prevalence of diabetes, with approximately 6% prevalence of undiagnosed diabetes [13]. A separate meta-analysis identified the prevalence of diabetes at 7.8% and prediabetes at 10.1% in the general population [14]. Although more recent national data on impaired fasting glucose (IFG) and impaired glucose tolerance (IGT) are unavailable, a 2009 study conducted in Dhaka reported the prevalence of IFG at 3.4%, IGT at 4.0%, combined IFG + IGT at 1.2%, and type 2 diabetes mellitus (T2DM) at 7.9% [15].

Therefore, the growing prevalence of diabetes and prediabetes presents a grave public health concern, potentially affecting a substantial portion of the working population in Bangladesh. Indeed, it has been reported that people’s occupation is associated with the onset of hyperglycemia and diabetes-related complications [16]. Professional drivers, who typically operate in the informal sector with long and irregular working hours, limited access to healthcare, and poor dietary and sleep conditions, are particularly vulnerable to unrecognized hyperglycemia and lack of diabetes-related health awareness [17], necessitating urgent attention.

In the socio-economic context of Bangladesh, professional drivers, often illiterate and neglected as working-class individuals, are excluded from societal welfare programs and policy considerations. Furthermore, while diabetes is a factor in medical fitness assessments for driving in developed countries [18,19], no such protocols exist in Bangladesh to monitor blood glucose levels in this workforce. To date, no documented studies have evaluated the prevalence of diabetes or the level of health awareness among professional drivers in the country. Driving, which is a cognitively and physically demanding occupation, requires precision, prolonged attention, quick reflex, and rapid decision-making, which can be impaired by both hyper and hypoglycemia, thereby posing significant risks to driving safety [20–22]. Studies have shown that hyperglycemia is associated with slower reaction times and diminished decision-making ability [20], while hypoglycemia can result in impaired judgment, delayed responses, and reduced concentration behind the wheel [21,22]. Therefore, raising awareness about diabetes and promoting routine blood glucose monitoring is crucial for improving drivers’ health outcomes and potentially reducing motor vehicle accidents associated with diabetes.

A Swedish nationwide study found professional drivers to be among the groups most susceptible to diabetes due to lifestyle factors such as diet, smoking, and poor sleep quality [23]. We hypothesized a similar scenario in Bangladesh, where nearly 20 people die each day because of fatal roadside crashes [24]. Consequently, a rapid, straightforward, and cost-effective blood glucose test is crucial for these drivers to reduce complications of diabetes, which are associated with road accidents [19]. Random Blood Glucose (RBG) screening, which measures blood glucose levels at any time of the day regardless of fasting status, has been endorsed by current guidelines for detecting undiagnosed hyperglycemia and improving diabetes screening [17,25]. According to the American Diabetes Association (ADA), an RBG level of ≥11.1 mmol/L (200 mg/dL) is indicative of diabetes when accompanied by classic symptoms of hyperglycemia [26]. An observational study in the U.S. demonstrated that random plasma glucose could be used opportunistically during outpatient visits to detect diabetes early [27]. Similarly, studies in resource-limited settings, such as Tanzania and India, utilized RBG to assess hyperglycemia prevalence, highlighting its feasibility when fasting blood glucose (FBG) testing is unavailable [28,29]. These studies demonstrate that RBG testing can be implemented opportunistically or in a case-finding approach to assess the prevalence of undiagnosed hyperglycemia, increase diabetes awareness, and identify risk factors in a resource-limited setting [25,27–30].

Our study utilized opportunistic RBG screening to evaluate the prevalence of undiagnosed diabetes and related awareness among professional drivers in northeastern Bangladesh, considering risk factors such as region, body weight, age, education level, and behavioral differences influenced by regional and cultural variations, which may contribute to both diabetes susceptibility and lower recognition of the condition. While factors such as body weight, age, behavioral patterns, and regional differences are associated with diabetes development [31,32], regional and educational disparities, particularly in rural populations, have been linked to low diabetes awareness and limited healthcare-seeking behavior, which may impact diabetes recognition [33,34]. In a country like Bangladesh, where no government-guided health policy addresses diabetes prevalence in the working-class population, our study is the first to fill this knowledge gap for professional drivers. While this study did not use Fasting Blood Glucose (FBG), Glycated Hemoglobin (HbA1c), or Oral Glucose Tolerance Test (OGTT) due to its opportunistic design and participants’ natural unwillingness to undergo intensive and time-consuming testing, it highlights the utility of RBG screening in detecting undiagnosed diabetes, providing actionable insights for policymakers to design inclusive national health strategies.

## Materials and methods

### Study design and participants

This cross-sectional study employed opportunistic random blood glucose (RBG) testing on professional drivers undergoing a government-mandated dope test, a prerequisite for obtaining a professional driving license, as per directives from the Bangladesh Road Transport Authority (BRTA). The tests were conducted at the Department of Biochemistry and Molecular Biology, Shahjalal University of Science and Technology (SUST), Sylhet-3114, Bangladesh, which serves as a designated site (approved by BRTA) for dope testing in the Sylhet region. This screening program was introduced to identify potential drug abuse and promote road safety in Bangladesh [35].

**Fig 1:**
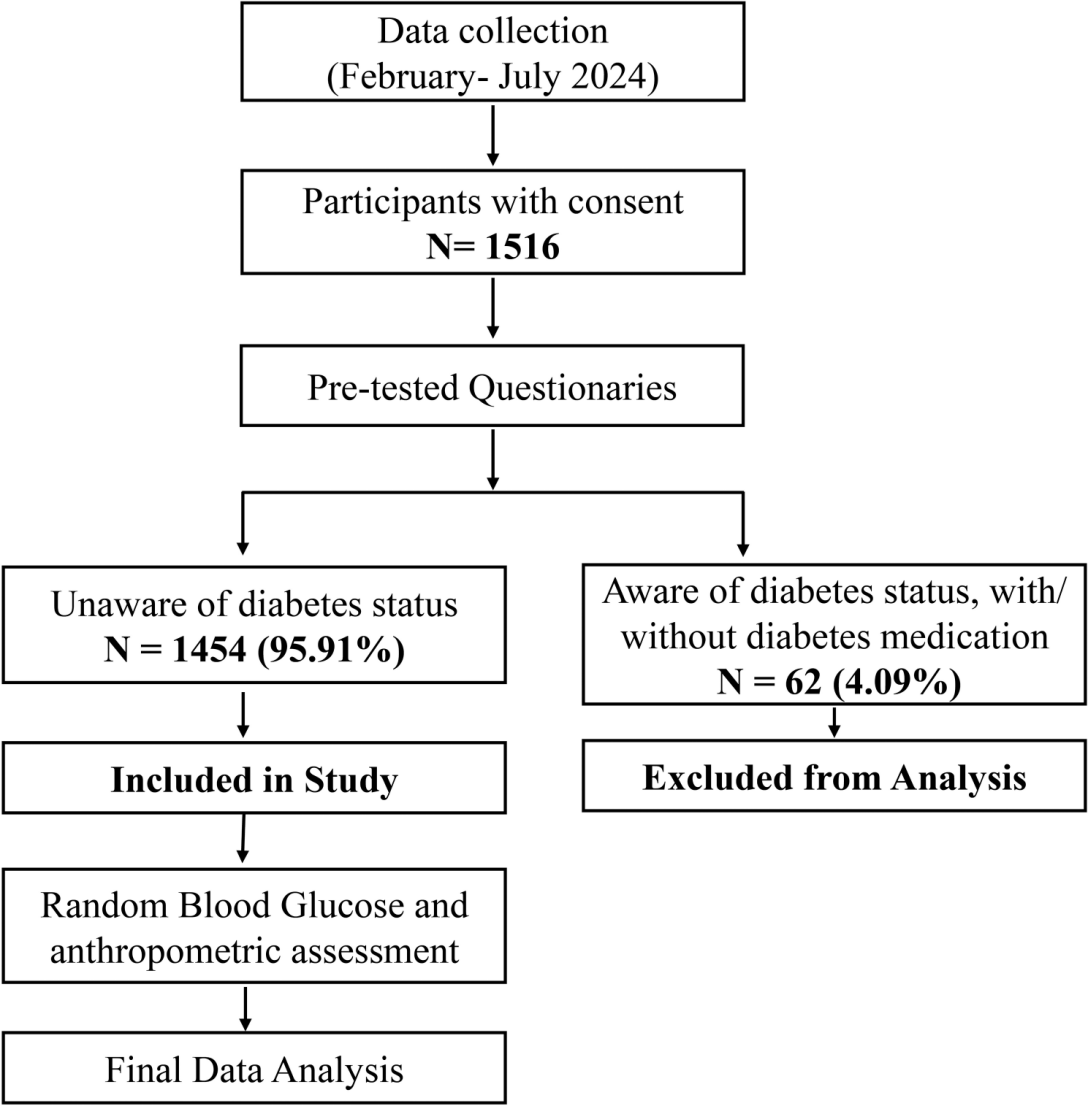
Flowchart depicting the study design, sampling, and exclusion criteria. Out of 1,516 participants who provided consent, 62 were excluded for those pre-diagnosed with diabetes or using medication affecting blood glucose levels.

In this context, all eligible participants were enrolled using a consecutive sampling approach between February 5, 2024 and July 27, 2024. Of the 1,516 participants who provided written consent, 1,454 were included in the final analysis, while 62 were excluded due to a prior history of diabetes, ongoing diabetes medication (e.g., insulin), or the use of medications that influence blood glucose levels (e.g., glucocorticoids and thiazide diuretics) to ensure the reliability of the findings as per standard screening protocols [26]. No formal sample size calculation was performed. Given the opportunistic and consecutive nature of the sampling, which was limited to drivers attending government-mandated dope tests at the SUST campus, our sample may not fully represent the broader professional driver population across Bangladesh. This may introduce a potential regional selection bias. The participants primarily represented four districts in the Sylhet division: Sylhet, Moulvibazar, Sunamganj, and Habiganj, while additional participants from other regions were categorized as ‘Others’. The sampling process and exclusions are detailed in Fig 1.

### Blood glucose testing and physical measurement

Random blood glucose levels were measured using an Accu-Chek Active glucometer (Model GB, Roche Diagnostics), which provides plasma-equivalent glucose values according to the manufacturer’s specifications. Before measurement, the participant’s fingertip was cleaned with an alcohol swab, and a disposable lancet was used to obtain a minimal amount of capillary blood on the fitted strip in the glucometer. Following the American Diabetes Association (ADA) guidelines, a random glucose level of ≥11.1 mmol/L was classified as diabetes [26].

Participant’s height was recorded to the nearest 0.1 cm using a stadiometer (Jiangsu Yuanyan Medical Equipment Co., Ltd.), while weight was measured to the nearest 0.1 kg using a calibrated digital electronic LCD weighing machine (Beurer PS 240, Germany), with participants wearing light clothing and no shoes. Body mass index (BMI) was calculated by dividing weight in kilograms by the square of height in meters (kg/m²). All RBG testing and anthropometric measurements were performed on-site by trained laboratory technicians and staff. All equipment was calibrated daily to ensure accuracy. Participants with elevated RBG levels were counseled to seek medical evaluation from certified physicians.

### Ascertainment of key variables and risk factors

Risk factors for diabetes were selected based on guidelines from the World Health Organization (WHO) and the Centers for Disease Control and Prevention (CDC), supported by an extensive literature review. Recognized risk factors included obesity, physical inactivity, age, family history of diabetes, dietary habits, and smoking.

Prior research has identified occupation-related risk factors such as disrupted sleep patterns [36], chronic stress [37], and unprescribed medicine intake indicative of drug abuse, which were evaluated in our study for their impact on blood glucose levels among the under-educated driver population [38]. To ensure data accuracy, alcohol intake was excluded from the study, as participants might withhold truthful responses during the mandatory dope test for license renewal.

Cultural and region-specific behaviors, such as betel quid and nut chewing, common in Northeastern Bangladesh due to the local cultivation of *Areca catechu* and *Piper betel*, were included in the study, as these behaviors have been significantly correlated with diabetes [39,40]. In this study, RBG levels were the dependent variable, with body mass index (BMI), age, sleep duration, smoking, family history, and other identified risk factors serving as independent variables to assess their variability and potential associations with blood glucose levels.

### Demographic and behavioral data collection

After enrollment, sociodemographic and lifestyle-related data were collected using pre-tested questionnaires (S1 Text) designed to capture relevant risk factors. Trained staff conducted interviews to gather information on participants’ location, sex, age, family history of diabetes, type of vehicle driven, educational status, diabetes knowledge, comorbidities, medication use, temper and stress-related concerns, smoking habits, driving duration and schedule, sleep duration and quality, and habitual consumption of betel nut (BN) and betel quid (BQ). Although the questionnaire was administered by trained staff to improve reliability, all behavioral variables (e.g., sleep, smoking, stress, medicine intake) were self-reported and may be subject to recall or social desirability bias.

To ensure accuracy, the staff carefully phrased questions to explicitly focus on “current behaviors,” defined as those occurring within the past 30 days, to assess their immediate effect on RBG levels. This approach aligns with established guidelines from the CDC’s Behavioral Risk Factor Surveillance System (BRFSS) and WHO’s STEPwise Approach to Surveillance (STEPS), ensuring consistency with globally recognized standards.

### Data collection and statistical analysis

Demographic, behavioral, and measurement data were collected using structured questionnaires administered by trained staff. The collected data were subsequently entered into Microsoft Excel spreadsheets (Microsoft Corporation, USA). All entries were carefully reviewed to ensure consistency with the original questionnaire responses and to correct any typographical errors. Statistical analyses were performed using SPSS (Statistical Package for Social Sciences) version 26 and GraphPad Prism 8 (GraphPad Software, Boston, USA), with graphic design completed in Adobe Illustrator 2022. After confirming the non-normal distribution of data through descriptive statistics and visual inspection, non-parametric tests were applied. Variations in RBG levels were assessed using the Mann-Whitney U test and Welch ANOVA, while linear relationships were evaluated using Spearman correlation.

Risk factors for binary logistic regression were selected through a stepwise approach based on significant associations identified in univariate analyses (Mann-Whitney U test and Spearman correlation). Binary logistic regression was then used to explore relationships between independent variables and the dependent variable, defined as diabetes status (with diabetes vs. without diabetes). A P-value less than 0.05 (p < 0.05) was considered statistically significant (*), while P-values greater than 0.05 (p > 0.05) were considered not significant (ns).

### Study approvals and participants’ consent

The study protocol was formally established following a rigorous evaluation by the SUST Research Ethics Board (SREB), Shahjalal University of Science and Technology (SUST), and received ethical approval (ref: DSLS-326) to conduct the research project. SREB is the only authorized organ to issue any ethical certificate concerning human subject research in SUST.

Each participant was thoroughly briefed on the objectives and aims of this study prior to enrollment. Subsequently, written informed consent was obtained from each participant. Additionally, participants were assured that all data gathered would be used exclusively for academic research, and any personal identifiers, including their names, would remain confidential.

## Results

### Baseline characteristics and regional and vehicle-wise categorization of the study population

**Fig 2:**
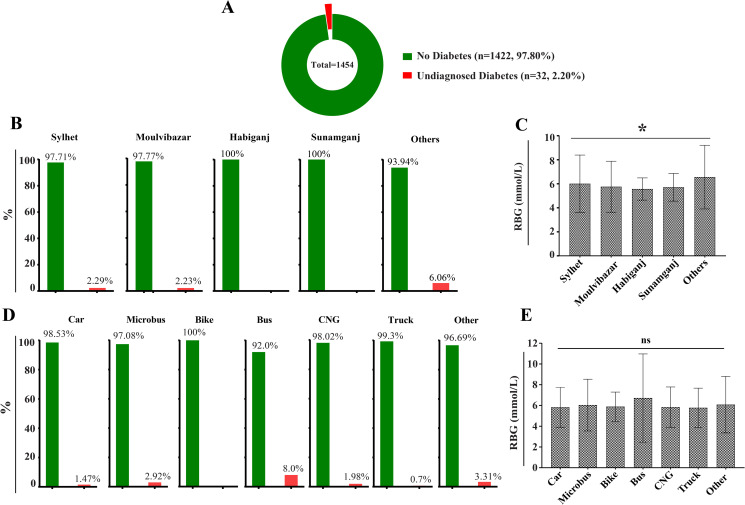
Distribution of the studied driver population into different subcategories. **(A)** Distribution based on the RBG levels: without diabetes (<11.1 mmol/L) and undiagnosed diabetes (≥11.1 mmol/L). **(B)** Regional distribution of the studied driver population across Sylhet (n = 918), Moulvibazar (n = 404), Habiganj (n = 31), and Sunamganj districts (n = 68) and other areas (n = 33) outside of Sylhet division **(C)** Comparison of mean RBG levels across different regional groups. **(D)** Proportion of participants based on their vehicle type: Car (n = 476), Microbus (n = 274), Bike (n = 26), Bus (n = 50), CNG (n = 305), Truck (n = 142), and Other (n = 181). **(E)** Comparative analysis of mean RBG levels across different vehicle driver groups.

To assess the prevalence of undiagnosed diabetes among professional drivers from northeastern Bangladesh, we screened their random blood glucose (RBG) levels at the Department of Biochemistry and Molecular Biology, SUST, Sylhet. Of the 1,454 participants, 97.80% (n = 1,422) had glucose levels below the diabetes threshold, while 2.20% (n = 32) were identified with undiagnosed diabetes without prior knowledge of their condition (Fig 2A). Participants without diabetes had a mean RBG level of 5.656 ± 1.18 mmol/L, whereas participants characterized by diabetes had significantly elevated levels (17.787 ± 5.19 mmol/L). The baseline characteristics of the studied population are presented in Table 1.

**Table 1:** Baseline characteristics of the study population.

Variables	Sub-group	n (%)
**Categorical**	**Regional location**	
	Sylhet	918 (63.14%)
	Moulvibazar	404 (27.79%)
	Habiganj	31 (2.13%)
	Sunamganj	68 (4.68%)
	Others	33 (2.27%)
	**Sex**	
	Male	1448 (99.6%)
	Female	6 (0.4%)
	**Family history of diabetes**	
	No	1260 (86.7%)
	Yes	194 (13.34%)
	**Level of Education**	
	Illiterate	31 (2.13%)
	Primary	315 (21.66%)
	Secondary	776 (53.37%)
	Higher Secondary	84 (5.78%)
	Graduate and Above	75 (5.2%)
	**Vehicle Type**	
	Car	476 (32.73%)
	Microbus	274 (18.84%)
	Bike	26 (1.65%)
	Bus	50 (3.43%)
	CNG	305 (20.9%)
	Truck	142 (9.8%)
	Other	181(12.45%)
	**Smoking Habit**	
	Yes	514 (35.35%)
	No	940 (64.65%)
	**Betel Quid Chewing Habit**	
	Yes	692 (47.59%)
	No	762 (52.4%)
	**Betel Nut Chewing Habit**	
	Raw	153 (10.52%)
	Mixed	238 (16.37%)
	Dry	422 (29.02%)
	No	641 (44.09%)
	**Sleep Quality**	
	Sound	773 (53.16%)
	Not Sound	61 (4.19%)
	Not Known	620 (42.56%)
	**Current Medication Intake (Non-Diabetes)**	
	Yes	132 (9.08%)
	No	1322 (90.92%)
	**Comorbidity**	
	Yes	29 (1.99%)
	No	1425 (99.01%)
	**Physical Exercise**	
	Yes	887 (61%)
	No	503 (34.59%)
	Not known	64 (4.4%)
**Continuous**	**Age (years)**	
	18-25, young adult (324, 22.28%)	23.262 ± 1.30
	26-44, mature adult (992, 68.23%)	33.347 ± 5.08
	45-59, middle age (131, 9.01%)	49.236 ± 3.90
	60 or above, old (7, 0.48%)	62.857 ± 4.29
	**Body Mass Index (kg/m**^**2**^)	
	<18.5, underweight (157, 10.79%)	17.289 ± 1.02
	18.5-24.9, healthy weight (844, 58.05%)	21.742 ± 1.84
	25-29.9, overweight (398, 27.37%)	26.964 ± 1.31
	≥30, obese (55, 3.78%)	31.482 ± 1.55
	**Random Blood Glucose (mmol/L)**	
	≤11.1, non-diabetic (1422, 97.80%)	5.656 ± 1.18
	>11.1, diabetic (unaware) (32, 2.20%)	17.787 ± 5.19
	**Sleep duration (hours)**	5.632 ± 0.72
	<7, short sleep duration (243, 16.71%)	
	≥7, healthy sleep duration (1211, 83.29%)	8.362 ± 1.31
**Total participants (n)**		**1454**

Regional variations in the studied driver population are illustrated in Fig 2B and 2C. Remarkably, participants from outside the Sylhet division (n = 33) showed the highest diabetes prevalence (6.06%), followed by Sylhet (2.29%) and Moulvibazar (2.23%) within this region (Fig 2B). Additionally, participants exhibited significant variation in RBG levels, likely attributed to the demographic characteristics of these regions (Fig 2C).

To examine associations between RBG levels and specific driving conditions, participants were categorized by vehicle type. Bus drivers (n = 50) had the highest proportion of individuals with undiagnosed diabetes (8.0%), followed by Microbus drivers (2.29%) and the ‘Other’ vehicle category (3.31%) (Fig 2D). However, ANOVA revealed no significant differences in mean RBG levels among driver groups (Fig 2E), suggesting that professional drivers may face a uniform risk of diabetes irrespective of vehicle type.

### Impact of age, BMI, and sleep duration on RBG level of the driver population

Age and body mass index (BMI) are critical factors that influence blood glucose levels. To examine variations in RBG levels, participants were categorized by age (young adults: 18–25 years, mature adults: 26–44 years, middle-aged: 45–59 years, and old: ≥ 60 years) and BMI based on WHO criteria (underweight: < 18.5 kg/m², healthy weight: 18.5–24.9 kg/m², overweight: 25-29.9 kg/m², and obese: ≥ 30 kg/m²). Additionally, participants were grouped by sleep duration (>7 hours: healthy sleep duration, < 7 hours: short sleep duration) following consensus recommendations [41].

**Fig 3:**
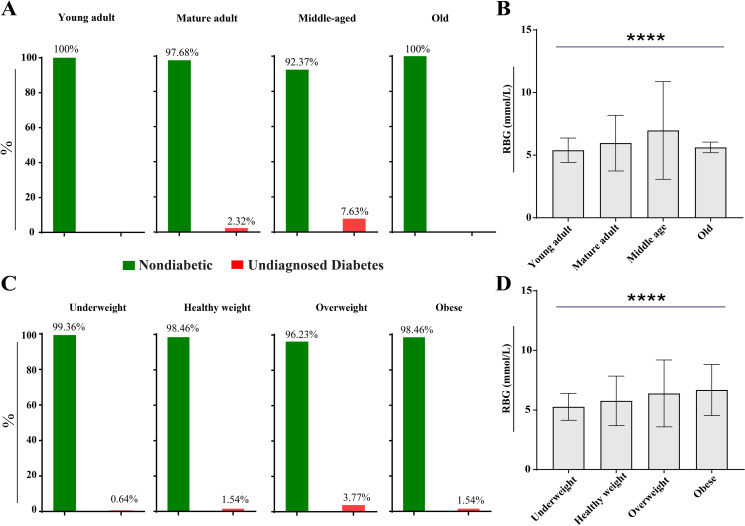
Relationship between Random Blood Glucose (RBG) levels, age, BMI, and sleep duration categories. **(A)** Distribution of RBG levels across age groups: young adult (n = 324), mature adult (n = 992), middle age (n = 131), and old (n = 7). **(B)** ANOVA comparison of mean RBG levels among age categories. **(C)** Frequency of participants, within different BMI groups: underweight (n = 157), healthy weight (n = 844), overweight (n = 398), and obese (n = 55). **(D)** ANOVA comparison of mean RBG levels among BMI categories.

Notably, the middle age group (n = 131) exhibited the highest proportion of participants with undiagnosed diabetes (7.63%), followed by the mature adult group (2.32%) (Fig 3A). No participants with diabetes were observed in the young adult and old groups, possibly due to the exclusion of individuals with pre-diagnosed diabetes from the analysis. A significant difference (P < 0.0001) in mean RBG levels was observed among age groups, indicating a strong association between RBG levels and age (Fig 3B).

In the context of BMI categories, 3.77% of the overweight population were found to have undiagnosed diabetes, followed by the obese (1.54%), healthy weight (1.54%), and underweight (0.64%) categories (Fig 3C). The ANOVA test further supported a statistically significant association (p < 0.0001) between RBG levels and BMI categories, indicating a relationship between elevated RBG levels and higher BMI (Fig 3D).

### Association of RBG with BMI, age, and sleep duration

**Fig 4:**
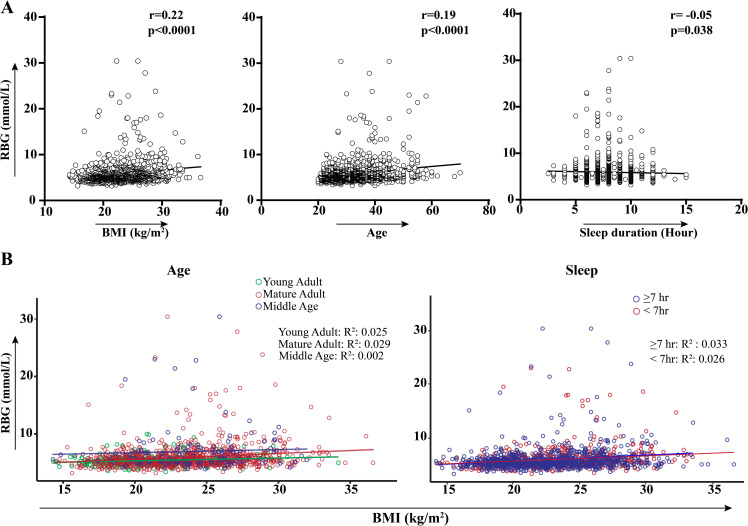
Correlation of RBG level with confounding variables BMI, age, and sleep duration. (A) Spearman’s correlation analysis shows a positive association between BMI and age with RBG levels and a negative correlation between RBG levels and sleep duration. (B) Multiple regression model assessing the interaction effect of BMI and RBG levels concerning age and sleep duration. R² denotes the proportion of variance in RBG levels explained by the independent variables (BMI, age, and sleep duration).

Our analysis identified statistically significant associations between RBG levels and confounding factors such as BMI and age. Spearman’s correlation analysis revealed a positive correlation between RBG levels and BMI (r = 0.22, p < 0.0001) and age (r = 0.19, p < 0.0001). Interestingly, RBG levels were negatively correlated with sleep duration (r = -0.05, p = 0.038), suggesting that increased RBG levels could potentially be linked to aging, increased body weight, and reduced sleep duration (Fig 4A).

To investigate the interactions among these confounding variables, a multiple linear regression model was developed (Fig 4B). Upon examining the overall association between BMI and RBG levels with respect to age, no statistically significant association was found, indicating that the relationship between RBG levels and BMI does not significantly vary across different age groups. BMI (coefficient, β = 0.047, p = 0.207) and age groups (mature adults: β = -0.807, p = 0.384; middle age: β = 1.311, p = 0.409) were not statistically significant independent predictors of RBG levels. Similarly, the interaction effects of BMI with mature adults (p = 0.206) and BMI with middle-aged adults (p = 0.938) were also insignificant, suggesting no meaningful modification of the BMI-RBG relationship by age.

For sleep duration, BMI was identified as a significant predictor of RBG levels in both sleep groups. For participants with less than 7 hours of sleep, BMI had a positive and significant association with RBG levels (β = 0.105, p = 0.011 R^2^ = 0.026). Similarly, for participants with 7 or more hours of sleep, BMI also had a positive and stronger association with RBG levels (β = 0.109, p < 0.001, R^2^ = 0.033). However, the overall model was not statistically significant (β = 0.005, p = 0.912), indicating that sleep duration does not modify the relationship between BMI and RBG.

### Association of RBG with lifestyle and anthropometric parameters

To examine the correlations between RBG levels and lifestyle factors, RBG levels were analyzed in relation to demographic and behavioral data, including family history of diabetes, comorbidities, medication use, smoking, betel quid and nut consumption, temperament, stress, sleep quality, and educational level.

**Fig 5:**
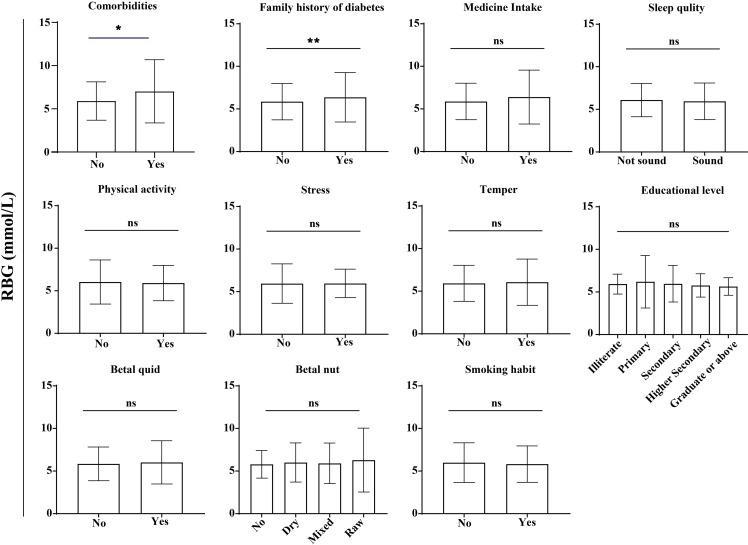
Association of RBG levels with various lifestyle parameters. Statistical analyses were conducted using the Mann-Whitney test and Welch ANOVA, with significance denoted as * (p < 0.05) and ** (p < 0.005); “ns” denotes not significant.

Notably, a family history of diabetes was significantly associated with higher RBG levels (Fig 5). Participants with a positive family history (n = 194) exhibited a higher mean RBG level compared to those without a family history of diabetes (n = 1,260) (6.37 ± 2.9 mmol/L vs. 5.85 ± 2.14 mmol/L, p < 0.005). Similarly, participants with comorbidities (n = 29) had significantly higher RBG levels than those without comorbidities (n = 1,425) (7.02 ± 3.66 mmol/L vs. 5.89 ± 2.24 mmol/L, p < 0.05). However, no significant associations were observed between RBG levels and other factors, including medication use (non-diabetic, non-glucose-affecting), sleep quality, smoking habits, stress, temperament, educational level, physical activity, or the consumption of betel quid and betel nut (Fig 5).

A binary logistic regression model was constructed to further explore the association between glucose levels and key predictors, including BMI, age, family history of diabetes, and comorbidities (Table 2). Binary logistic regression could not be applied to age groups when comparing mature adults, middle-aged, and older categories to young adults as a reference group, as no participants in the latter group had identified with diabetes (RBG < 11.1 mmol/L).

**Table 2:** Binary Logistic Regression Model for RBG as a Predictor of Diabetes Status. RBG is the dependent variable, categorizing participants into two groups: without diabetes (RBG < 11.1 mmol/L, n = 1,422) and with diabetes (RBG ≥ 11.1 mmol/L, n = 32).

Variables	Adjusted Odds Ratio (AOR)	95% Confidence Interval (CI)	P Value
**Age Groups**			
18-25 years, young adult (n = 324)^*^	Ref	Ref	Ref
26-44 years, mature adult (n = 992)	–	–	–
45-59 years, middle age (n = 131)	–	–	–
60 years or above, old (n = 7)^*^	–	–	1
**BMI**			
Healthy Weight (18.5-24.9, kg/m^2^) (n = 844)	Ref	Ref	Ref
Underweight (<18.5, kg/m^2^) (n = 157)	0.53	0.07 - 4.14	0.540
Overweight (25-29.9, kg/m^2^) (n = 398)	1.81	0.83 - 3.99	0.136
Obese (≥ 30, kg/m^2^) (n = 55)	3.04	0.81 - 11.46	0.100
**Comorbidity**			
No (n = 1425)	Ref	Ref	Ref
Yes (n = 29)	0.56	0.06 - 4.78	0.596
**Sleep (Hours)**			
≥ 7 hours (n = 1211)	Ref	Ref	Ref
< 7 hours (n = 243)	1.13	0.46 - 2.75	0.786
**Family History of Diabetes**			
None (n = 1260)	Ref	Ref	Ref
Father (n = 55)*	–	–	–
Mother (n = 94)	0.45	0.06 - 3.38	0.435
Parents (n = 21)	8.53	2.16 - 33.59	0.002
Parent/siblings (n = 10)	19.21	2.97 - 124.17	0.002
Siblings (n = 14)	2.27	0.27 - 18.10	0.449

* *indicates categories that had no participants with diabetes (RBG ≥ 11.1 mmol/L), making binary logistic regression analysis inapplicable for these groups.*

Among BMI categories, the obese group had the highest risk, with an adjusted odds ratio (AOR) of 3.04 (95% CI: 0.81–11.46), followed by the overweight group (AOR: 1.81, 95% CI: 0.83–3.99) compared to the healthy weight group. While these findings suggest that obese individuals are approximately three times more likely to have elevated RBG levels compared to those of healthy weight, the association did not reach statistical significance.

Participants with both parents and siblings with a history of diabetes had 19 times higher odds of elevated RBG levels compared to those with no family history (AOR: 19.21, 95% CI: 2.97-124.17, p = 0.002). Those with only parents with a history of diabetes had 8 times higher odds (AOR: 8.53, 95% CI: 2.16–33.59, p = 0.002).

In contrast, participants with a sleep duration of less than 7 hours had slightly higher odds of elevated RBG levels compared to those with a sleep duration of 7 hours or more (AOR: 1.13, 95% CI: 0.46–2.75, p = 0.786), but this association was not statistically significant.

## Discussion

Diabetes has become a major public health concern in lower-middle-income countries (LMIC) like Bangladesh, affecting a considerable portion of the working population. Despite its prevalence, government policies remain inadequate, and large segments of the population remain unaware of their diabetic or hyperglycemic status [42]. Professional drivers constitute a high-risk population for diabetes due to their limited physical activity while sitting behind the wheel [43]. In countries like Bangladesh, they often represent a neglected segment of the population, lacking essential health education and leading an unhealthy lifestyle, which contributes to higher rates of diabetes [11,44]. Furthermore, diabetes prevalence and practices related to blood glucose monitoring among these drivers are not routinely assessed, highlighting a significant knowledge gap in national health and road safety policies.

Like other LMICs, Bangladesh is experiencing a significant rise in non-communicable diseases (NCDs), with diabetes prevalence increasing by 1.1 times between 2011 and 2017, posing a major challenge to the country’s already strained healthcare system, despite government efforts to develop NCD surveillance guidelines [45]. Strengthening the health system for effective NCD management requires addressing several persistent issues, including the absence of standardized protocols, a shortage of trained healthcare professionals, limited laboratory infrastructure, restricted availability of essential medicines and logistics, and weak data recording and reporting mechanisms [46]. Despite the physically and cognitively demanding nature of driving, there is no government-mandated pre-employment or annual medical screening for professional drivers in Bangladesh that includes diabetes assessment. Prolonged sedentary behavior, irregular working hours [47], and limited access to healthy food options may further elevate diabetes risk in this population [48,49]. Incorporating routine metabolic health checks and occupational health interventions could improve disease detection and management among drivers. In this context, our findings highlight the value of integrating opportunistic blood glucose screening into existing community-based health programs, particularly targeting underserved, working populations. Enhancing primary healthcare infrastructure to support early detection, training frontline health workers in NCD screening, and expanding national diabetes awareness initiatives to include high-risk occupational groups, such as professional drivers, could be effective strategies for improving NCD prevention and control in Bangladesh. Thus, this cross-sectional study included 1,454 professional drivers from northeastern Bangladesh to evaluate their diabetes status and awareness using opportunistic RBG testing followed by questionary-based interviews, after obtaining proper written consent. Notably, 2.20% (n = 32) of our studied driver population was found to have diabetes (RBG ≥ 11.1 mmol/L) and were completely unaware of their hyperglycemic status (Fig 2A). This lack of diabetes knowledge and awareness poses significant health risks and threatens road safety, as diabetes-related consequences of drivers have been linked with increased road accidents [17,50]. A significant regional variation in RBG levels was observed among participants (Fig 2C), potentially attributed to differences in lifestyle and sociodemographic factors. This finding aligns with a study by Feng et al., which focused on rural areas in South Asian countries [51]. Additionally, diabetes prevalence and awareness showed regional variation, with drivers from outside of the Sylhet division having the most previously undiagnosed diabetes cases (6.06%) (Fig 2B).

When categorizing the studied driver population based on their vehicle types, bus drivers were at the highest risk of developing diabetes as 8.0% of them were unaware of their elevated glucose levels (Fig 2D). Further, they showed a higher mean RBG level (6.7 ± 4.2 mmol/L) compared to other groups, which might be associated with nighttime driving and reliance on roadside food, responsible for high glycemic conditions [52]. This result aligns with studies that link extended periods of driving under stressful and physically fatiguing conditions are associated with elevated RBG levels, which is considered one of the factors of roadside fatalities [53,54].

Our analysis revealed a statistically significant difference (p < 0.0001) in RBG levels among various age categories. The middle age group (45–59 years) exhibited the highest mean RBG level (6.98 ± 3.9 mmol/L), followed by the mature adult group (5.97 ± 2.2 mmol/L) and the old group (5.61 ± 0.42 mmol/L) (Fig 3B). These findings are consistent with a study in Ethiopia, which demonstrated that participants aged > 45 years are more likely to develop diabetes [55]. However, this result contrasts with a study from Iran, which showed that the prevalence of hyperglycemia increased progressively with age [56]. This may be attributed to the exclusion of individuals with pre-diagnosed diabetes (n = 62) from the analysis, most of whom belonged to the old category due to their frequent diagnoses stemming from worsening health conditions.

The study also identified body mass index (BMI) as a significant factor affecting RBG levels (Fig 3D). Overweight (3.77%) and obese (2.54%) categories had the highest percentage of participants with undiagnosed diabetes, indicating that weight significantly contributes to elevated glucose levels, consistent with findings from various studies [23,57]. This may also indicate that lacking health awareness may correlate with gaining weight and elevated glucose levels (Fig 3C).

Since our analysis revealed significant variations in RBG levels with respect to age and BMI, we utilized the Spearman correlation to explore this association (Fig 4A). Both age (r = 0.22, p < 0.0001) and BMI (r = 0.19, p < 0.0001) were positively correlated with RBG levels, suggesting that glucose levels slightly increased with age and body weight. These factors may contribute as potential risk factors for progressive hyperglycemia in Bangladeshi individuals [1]. Additionally, our binary logistic model indicated that obese individuals are approximately three times more likely to develop diabetes compared to those of healthy weight (AOR:3.04, 95% CI: 0.81-11.46), aligning with a study in the United States [58] (Table 2).

Previous research on the United Kingdom’s professional drivers has shown that disrupted sleep patterns, a common occupational hazard due to overtime or nighttime driving, are significantly associated with an increased risk of diabetes [36,52,59]. Consistent with this, our analysis found a negative association between sleep duration and RBG levels (r = -0.05, p = 0.38) (Fig 4A), suggesting that inadequate sleep may contribute to elevated glucose levels. Binary logistic regression also indicated that persons with short sleep duration (<7 hours) are 1.13 times more likely to have elevated glucose levels than participants with healthy sleep duration (≥7 hours) (Table 2).

Lifestyle parameters were significantly associated with RBG level and potential diabetes risk were identified as family history of diabetes and comorbidities. Notably, a positive family history of diabetes significantly increased susceptibility to hyperglycemia [60], with individuals having both parents and siblings with diabetes showing a 19.21 times higher likelihood (AOR: 19.21; 95% CI: 2.97-124.17). This finding aligns with a study in Ethiopia, which reported that individuals with a family history of diabetes were four times more susceptible [55]. Moreover, previous research supports the associations between RBG levels and comorbidities in populations from Bangladesh and Taiwan, which is consistent with our study [61]. Interestingly, our research did not find a significant correlation between smoking and hyperglycemia, consistent with a study on the Ethiopian population [55].

Our study highlights the prevalent issue of undiagnosed hyperglycemia among professional drivers in Bangladesh, reflecting a broader lack of diabetes awareness and health knowledge within the general working population. This study also aimed to raise awareness among drivers, as those identified with diabetes were counseled to undergo regular health checkups at professional healthcare providers. While our study draws attention to this often neglected group and highlights the importance of regular blood glucose monitoring, this is limited to measuring only random blood glucose due to the study conditions and opportunistic nature of this study. Blood pressure measurements, detailed dietary habits, oral glucose tolerance, and follow-up results should generally be included as RBG tests can be unpredictable and influenced by recent food or beverage intake, requiring careful interpretation of results. In contrast, Fasting Blood Glucose (FBG), Glycated Hemoglobin (HbA1c), and Oral Glucose Tolerance Test (OGTT) are considered gold-standard diagnostic tools due to their higher sensitivity and specificity. According to the American Diabetes Association, diabetes is diagnosed using FBG ≥ 7.0 mmol/L, OGTT ≥ 11.1 mmol/L, or HbA1c ≥ 6.5%. While RBG and Postprandial Blood Glucose (PPBG) tests share the same diagnostic threshold (≥ 11.1 mmol/L), they are generally less specific and used for preliminary screening or glucose monitoring, requiring confirmatory testing [26]. Due to logistical challenges and feasibility constraints related to the movable nature of the driver population, we were unable to use the gold-standard tests in our study Furthermore, the majority of participants (n = 992) belonged to the mature adult age group (26–44 years), as they are the most actively employed drivers. This may have influenced the overall average blood glucose levels and the proportion of undiagnosed diabetes cases (7.63%) observed in this group, potentially introducing a selection bias and requiring careful interpretation. However, these limitations do not diminish the importance of identifying undiagnosed diabetes in this high-risk population. For this purpose, RBG testing can serve as a valuable tool for opportunistic hyperglycemia screening in clinical practice and is widely recognized for its effectiveness [62,63]. The prevalence of undiagnosed diabetes (2.20%) identified in this study should be considered when formulating national health policies in Bangladesh. While Bangladesh has already implemented the WHO-supported Multisectoral Action Plan for Prevention and Control of Noncommunicable Diseases 2018–2025 [64] to address conventional NCDs, this strategy does not specifically address the health outcomes of underrepresented populations such as professional drivers. Additionally, the *Non-Communicable Disease Control Programme*, initiated by the Directorate General of Health Services (DGHS), has established dedicated NCD corners in Upazila Health Complexes to promote NCD prevention, treatment, and control [46]. However, to include the welfare of Bangladesh’s professional drivers, targeted mass awareness campaigns are needed to encourage their participation in routine diabetes screening. Simultaneously, stricter enforcement of the Road Transport Act 2018, which mandates seatbelt use, helmet requirements, and other safety provisions, is essential for reducing road traffic injuries. Broader factors influencing road safety, such as poor infrastructure, substandard vehicle safety, and inconsistent enforcement must also be addressed to protect both the long-term health of drivers and the safety of Bangladesh’s roads.

## Conclusion

Our study sheds light on a significant knowledge gap regarding blood glucose levels and the possible complications associated with diabetes among professional drivers from northeastern Bangladesh. This is emphasized by the finding that 32 out of 1,454 participants (2.2%) exhibited a random blood glucose (RBG) level above the diabetic threshold (≥ 11.1 mmol/L), yet were completely unaware of their condition prior to enrollment in our study. Due to its ease of administration, affordability, and minimal requirement for specialized equipment, opportunistic RBG screening could serve as a practical and viable option for large-scale implementation, as demonstrated by this pilot study. Policymakers can leverage these findings as a roadmap to design and implement effective screening programs, ensuring widespread coverage, early detection of diabetes, and improved health outcomes within the working population of Bangladesh. Integrating health education with such screening programs could further enhance awareness and promote preventative measures among high-risk groups.

## Supporting information

S1 Text Questionnaire form. (PDF)

S1 Data Anonymized dataset. (XLSX)
