# Supplementary material for: Opportunistic random blood glucose screening among professional drivers in northeastern Bangladesh: Assessing undiagnosed diabetes and health awareness
Source: PLOS Glob Public Health. 2025 Jun 24;5(6):e0004828. doi: 10.1371/journal.pgph.0004828 (PMC12186904; doi:10.1371/journal.pgph.0004828)
Supplement: S1 Text — (PDF) [file pgph.0004828.s001.pdf]

Department of Biochemistry & Molecular Biology
Shahjalal University of Science & Technology,
Sylhet -3114, Bangladesh.
Phone # PABX: (0821)- 713491/714479/
713850 /717850 Ext.- 409
Fax: 880-821-715257
E-mail : bmb@sust.edu

বায়োকেমিস্ট্রি এন্ড মলিকুলার বায়োলজি বিভাগ
শাহজালাল বিজ্ঞান ও প্রযুক্তি বিশ্ববিদ্যালয়,
সিলেট-৩১১৪,বাংলাদেশ|
ফোন # পিএবিএক্স (০৮২১)- ৭১৩৪৯১/
৭১৪৪৭৯/৭১৩৮৫০/ ৭১৭৮৫০-৪০৯
ফ্যাক্স নং : ৮৮০-৮২১-৭১৫২৫৭
ই-মেইল ঃ bmb@sust.edu

Title of the project:

Random blood sugar screening among the professional driver and assess the health awareness from northeastern part of Bangladesh.

Circle the appropriate answer to each of the following

Patients ID: **Date:**

Name of the patient:

1. Sex : (a) Male (b) Female
2. Age :
3. Blood sugar level (mmol/L) :
4. Occupation (type of vehicle) :
5. Family History of Diabetes : (a) Mother (b) Father (c) Siblings
6. Body weight (Kg) :
7. Height (cm):
8. Habit : (a) Walking (b) Sedentary lifestyle
9. Smoking : (a) Yes……….. (b) No
10. Betel Quid : (a) Yes (b) No
11. Betel Nut (Supari) : (a) Raw (b) Dry (c) No

12.Current Medicine:

13. Comorbid disease: (a) Yes **[ ]** b) No
14. Driving Hour: (a)………………. (b) D/N

15.Gross Sleeping Hour/Quality/Shift: (a, Hours) (b, Quality) Sound/Not Sound
(c) Day/Night

17. Education level:
18. Temper: (a) Yes ….. ....(b) No
19. Stress: (a) Yes……………………… (b) No
20. License: (a) Renew (b) New
21. Location:
